# Delayed Diagnosis of Chronic Q Fever and Cardiac Valve Surgery

**DOI:** 10.3201/eid1905.120353

**Published:** 2013-05

**Authors:** Linda M. Kampschreur, Elske Hoornenborg, Nicole H. M. Renders, Jan Jelrik Oosterheert, Joost F. Haverman, Peter Elsman, Peter C. Wever

**Affiliations:** University Medical Center Utrecht, Utrecht, the Netherlands (L.M. Kampschreur, J.J. Oosterheert);; Jeroen Bosch Hospital, ‘s-Hertogenbosch, the Netherlands (L.M. Kampschreur, E. Hoornenborg, N,H.M. Renders, J.F. Haverman, P. Elsman, P.C. Wever)

**Keywords:** Q fever, chronic Q fever, Q fever endocarditis, valvulopathy, Coxiella burnetii, cardiac surgical procedures, bacteria, zoonoses, valvular heart disease

## Abstract

Untreated chronic Q fever causes a high number of complications and deaths. We present cases of chronic Q fever that were not diagnosed until after the patients underwent cardiac valve surgery. In epidemic areas, Q fever screening of valve surgery patients secures early initiation of treatment and can prevent illness and death.

Q fever, a zoonosis caused by the intracellular gram-negative bacterium C*oxiella* *burnetii*, occurs in outbreaks and is prevalent worldwide. Q fever has acute and chronic stages (*1*). Acute Q fever is a self-limiting febrile disease occurring in 40%–50% of *C. burnetii*–infected persons (*1*). Chronic Q fever can develop years after primary infection and occurs in 1%–5% of *C. burnetii*–infected persons (*1*,*2*). The most critical manifestations of chronic Q fever are endocarditis and infections of vascular prosthesis and aortic aneurysms (*3*). Persons with pre-existing valvular cardiac disease have a reported 40% risk of Q fever endocarditis when infected with *C. burnetii* (*2**,**4*). 

During 2007–2010, an outbreak of >4,000 cases of acute Q fever occurred in the Netherlands (5). To increase understanding of the role of Q fever in valvular cardiac disease, we present 3 cases of chronic Q fever and valvular cardiac disease requiring surgery in patients from the Netherlands. The diagnosis of chronic Q fever was not made until after the patients had elective cardiac valve surgery for progressive valvular dysfunction.

## Case 1

In 2004, aortic valve stenosis of a tricuspid valve was diagnosed in a 73-year-old man. Additional medical history included atrial fibrillation and transient ischemic attacks. Because of progressive stenosis, the patient underwent aortic valve replacement with a bioprosthesis in May 2011. The removed valve had no macroscopic signs of endocarditis, so neither microbiological nor pathological examination was requested. Four months later, paravalvular insufficiency of the bioprosthesis developed in the patient, requiring a second valve replacement. Transesophageal echocardiography revealed no vegetations. Macroscopic signs of endocarditis were not observed on the removed valve; further examination was not requested. However, serologic testing for *C. burnetii* revealed chronic infection (Table). 

**Table:** Results of *Coxiella burnetii* and C-reactive protein testing for 3 patients with chronic Q fever and valvular cardiac disease requiring valve surgery, the Netherlands*

Case-patient	Results for *C. burnetii* testing			C-reactive protein, mg/L
	IFA†		PCR	
	Phase I IgG titer	Phase II IgG titer		
1				
Before valve surgery†	32,768	65,536	Negative	<6
After valve surgery	8,192	8,192	Positive	52
2				
Before valve surgery	16,384	16,384	Positive	22
After valve surgery	16,384	16,384	Positive	26
3‡				
After valve surgery	8,192	8,192	Positive	11

*IFA, immunofluorescence assay. †Q fever serologic testing was performed retrospectively. ‡Serologic testing was not available before surgery.

The patient had not been aware of previous acute Q fever infection and had not experienced fever, night sweats, weight loss, or malaise. Further examination by fluorodeoxyglucose positron emission tomography (FDG PET) combined with low-dose computed tomography (CT) demonstrated no other chronic Q fever focus or vascular abnormalities. The patient started antimicrobial drug therapy (doxycycline and hydroxychloroquine) and was doing well 3 months later. Retrospective microbiological examination of a serum sample obtained at the time of the first valve replacement demonstrated a profile consistent with chronic Q fever (Table).

## Case 2

A 78-year-old man had a medical history of aortic valve stenosis of a tricuspid valve, abdominal aortic aneurysm, and endovascular aneurysm repair in 2005. In July 2011, he was screened for chronic Q fever in a program for patients at high risk for development of chronic Q fever (e.g., persons with a vascular prosthesis or aneurysm) (*6*); the screening revealed that he did have chronic Q fever infection (Table). The patient had not been aware of an acute Q fever episode and did not report night sweats, weight loss, malaise, or fever. Because the he had progressive aortic valve stenosis, the patient was on a waiting list for elective valve replacement at an academic cardiovascular center. This center, located outside the Q fever epidemic area and unaware of the patient’s Q fever status, placed a bioprosthesis in the patient in August 2011. The native valve was not further examined because there were no macroscopic signs of endocarditis. 

After the surgery, the Q fever screening results were acted upon. FDG PET/CT scan results showed no signs of infection at the abdominal aortic prosthesis or elsewhere. In September 2011, the patient started antimicrobial drug therapy (doxycycline and hydroxychloroquine) and was doing well at a 6-month follow-up visit.

## Case 3

A 70-year-old woman had a longstanding history of rheumatoid arthritis that was treated consecutively with infliximab and etanercept plus corticosteroids and azathioprine. In 2009, she was hospitalized because of heart failure caused by mitral valve insufficiency, possibly resulting from chordal rupture, combined with an atrial septal defect and left ventricular systolic dysfunction. In October 2010, mitral valve repair, a coronary bypass, and atrial septal defect closure were performed. The patient was registered for vaccination against *C. burnetii*, which was offered by the government to persons with aortic (endo)vascular prostheses or cardiac valve abnormalities. In April 2011, prevaccination screening results showed she was positive for chronic Q fever (Table). The patient did not recall a previous acute Q fever episode, and she had not experienced fever, night sweats, malaise, or weight loss. FDG PET/CT scan results showed no FDG uptake in the large vessels. 

Transesophageal echocardiography revealed an insufficiency of the mitral valve repair. An echocardiogram was not performed immediately after the valve repair in 2010, so it could not be determined whether this insufficiency was new. No vegetations or signs of endocarditis were seen. Antimicrobial drug treatment (doxycycline and hydroxychloroquine) was started and later switched to moxifloxacin monotherapy because of elevated liver enzyme levels and severe nausea and vomiting, possibly caused by hydroxychloroquine. After 15 months of treatment, the patient still had a high level of *C. burnetii* antibody.

## Conclusions

We reviewed 3 cases of chronic Q fever and valvular cardiac disease requiring surgery. The diagnosis of chronic Q fever was not made until after the elective surgery. Early diagnosis and antimicrobial drug treatment of Q fever endocarditis might have prevented surgery. Symptoms of Q fever endocarditis can be nonspecific, and vegetations are usually absent or small (*7*). As observed in the cases presented here, C-reactive protein levels can be normal or only mildly elevated (*8*) (Table). The most frequent signs of Q fever endocarditis are a new valvular insufficiency or worsening of preexisting valvular insufficiency (*8*–*10*). *C. burnetii*–infected cardiac valves can appear normal on visual inspection, as demonstrated in the cases presented here, and on histologic evaluation (*11*). 

Diagnosis of chronic Q fever is challenging. Chronic infection is determined on the basis of serologic testing and PCR of blood samples and, if available, tissue samples. In the absence of acute Q fever, PCR results positive for *C. burnetii* in blood or tissue prove chronic infection; however, the sensitivity of this test is only 50%–60% in patients with chronic Q fever (*12*). When cultured in cells, *C. burnetii* exhibits antigenic variation in which the virulent variant, called phase I, shifts to an avirulent variant, called phase II. During acute infection, antibodies to phase II antigens are detected first; persisting high levels of antibodies to phase II, and especially phase I antigens, are indicative of chronic Q fever (*13*). A phase I IgG titer >800 or >1,024, depending on the type of immunofluorescence assay used, has been internationally accepted for the serologic diagnosis of chronic Q fever (*14*,*15*). 

Long-term antimicrobial drug treatment, preferably doxycycline plus hydroxychloroquine, is the treatment of choice for chronic Q fever. Treatment should continue for 18 months for native valves and 24 months for prosthetic valves, until a 4-fold decrease of phase I IgG titers and a complete clearance of phase II IgM are reached. If phase I IgG titers remain high or phase II IgM is detectable, treatment should be extended. The rates of morbidity and mortality among people with chronic Q fever are high, reaching >60% if treatment is delayed or not initiated. With adequate treatment, the mortality rate for Q fever endocarditis has declined to 5%. Chronic Q fever involving prosthetic valves is associated with a higher mortality rate, longer treatment, and elevated chance of complications (*9*). For the cases reported here, preoperative diagnosis of chronic Q fever might have prevented the second valve replacement in case-patient 1 and the delay in treatment initiation in case-patients 2 and 3. We advise preoperative serologic screening for chronic Q fever in all patients undergoing elective cardiac valve surgery in Q fever epidemic areas. If serologic test results are positive for *C. burnetii* antibodies, PCR of the excised valve should be performed.
